# Digital social media expression and social adaptability of the older adult driven by artificial intelligence

**DOI:** 10.3389/fpubh.2024.1424898

**Published:** 2024-08-29

**Authors:** Yuan Gao, Jiahui Liang, Zhengbing Xu

**Affiliations:** ^1^Advanced Graduate School of Imaging, Chung-Ang University, Seoul, Republic of Korea; ^2^School of Art, Hubei University, Wuhan, China; ^3^School of Art and Design, Wuhan Technology and Business University, Wuhan, China; ^4^Local Art, Udon Thani Rajabhat University, Udon Thani, Thailand

**Keywords:** artificial intelligence-driven, digital new media art expression, older adult individuals, social adaptability, short videos

## Abstract

**Introduction:**

This study examines the impact of digital new media art on the health literacy and digital health literacy of older adults. It explores how digital new media art influences the social adaptability of the older adult, with a focus on variations in their engagement with digital technologies and community activities.

**Methods:**

The research employed interviews and observations of older adult participants from communities *A* and *B*. Data were collected on their smartphone usage, community engagement, and access to technological infrastructure. The study also assessed their interaction with digital new media across various domains, including interpersonal communication, information retrieval, entertainment, practical applications, and mobile payments.

**Results:**

The study found significant differences in engagement with digital new media art among the older adult. Participants with prior computer experience were generally more skilled in using smartphones and more active in community events. In contrast, individuals in community *B* showed lower acceptance of digital new media art and no clear association with community participation. There was substantial variability in their use of digital media for information retrieval, entertainment, practical applications, and mobile payments. Some older adult individuals demonstrated proficiency with these technologies, while others were more reserved.

**Discussion:**

The findings suggest that digital new media art can enhance community participation and social adaptability among older adults, particularly those with prior computer experience. However, disparities in digital media usage highlight the need for targeted interventions to improve digital health literacy and engagement across different community settings. The study underscores the importance of addressing these disparities to ensure that all older adults can benefit from digital advancements, thereby improving their overall well-being and health literacy.

## 1 Introduction

With the rapid development of society and technological progress, artificial intelligence (AI) technology has permeated various aspects of life. Whether in industrial production, healthcare, transportation, or daily life, AI technology plays an increasingly crucial role (1, 2). In the field of art, digital new media art is gaining attention and popularity, becoming a new channel and form of self-expression. Encompassing various forms such as digital painting, virtual reality, and interactive installations, digital new media art, based on digital technology, transcends the limitations of traditional art forms, providing new possibilities for artistic creation and expression (3, 4).

As technology continues to advance, digital new media art exhibits a progressive character. Notably, the application of AI technology injects new vitality and creativity into digital art (5). Through techniques like machine learning and deep learning, AI can simulate human creativity and imagination, producing astonishing works of art (6–9). Concurrently, with the evident trend of an aging population, issues related to the social adaptability of older adult individuals have become a focal point of societal concern (10). Older adult individuals face challenges such as declining physical health, shifts in social roles, and changes in family structure, necessitating adaptation to new living environments and social roles (11). In this process, art, as a cultural and spiritual activity, holds significant social functions and meaning. Through participation in artistic creation and appreciation, older adult individuals can enrich their spiritual lives, enhance social adaptability, alleviate stress and loneliness, and improve overall quality of life. Digital new media art, as an emerging art form, provides older adult individuals with novel artistic experiences and social interaction methods (12). Firstly, digital new media art exhibits characteristics of diversity and interactivity, allowing older adult individuals to engage in digital art creation and appreciation, communicate with others, strengthen social connections, and reduce feelings of loneliness. Secondly, as a creative activity, digital new media art can stimulate the creativity and imagination of older adult individuals, enhancing their sense of self-identity and confidence, thereby facilitating better adaptation to the social environment (13, 14). Additionally, digital new media art can offer a diverse range of spiritual enjoyment, enhancing the overall quality of life and promoting mental and physical well-being.

This study focuses on the theme of the expression of digital new media art and social adaptability, targeting the older adult population, to explore the impact of AI-driven digital new media art expression on the social adaptability of older adult individuals. The ability of older adult individuals to adapt to technology determines their ability to adapt to the changing times. This study aims to advocate for community management that aligns with a “people-centered” philosophy and standards, recognizing the adaptive needs of older adult individuals, and emphasizing human care.

## 2 Literature review

As the global trend of aging intensifies, the social adaptability and mental health of the older adult have garnered increasing attention. Social media and communication technologies, as vital means of modern information exchange, have become focal areas of research regarding their impact on the quality of life and mental health of the older adult. This review summarizes and analyzes several significant studies conducted in recent years, exploring the role of social media and communication technologies in enhancing the social adaptability, information usage, mental health, and social connections of the older adult. This review aims to provide a theoretical foundation and practical guidance for further research and practice. Chen and Gao (15) investigated the effects of social media self-efficacy on the information usage, loneliness, and self-esteem of the older adult. Through a survey of 276 Chinese older adult individuals aged 60–90 and the construction of a structural equation model, higher social media self-efficacy was closely associated with increased information usage, reduced loneliness, and higher self-esteem. While information usage reduced loneliness, it did not significantly impact self-esteem. This indicated that enhancing social media self-efficacy in the older adult was crucial for improving their mental health and social adaptability (15). Wilson et al. (16) adopted a qualitative, exploratory approach, conducting semi-structured interviews with 20 older adult individuals (aged 65 and above) in England, Scotland, and Wales to examine their experiences with social technologies. Although the older adult could maintain social connections through social technologies, various barriers affected their motivation and skills to use these technologies. These barriers included perceived self-efficacy and fear, online communication culture, lack of social capital, and physical limitations. These obstacles, while similar to those encountered with general technology use, also presented unique challenges specific to social technology use. This highlighted the need to consider these factors when providing guidance and interventions to increase online social connections among the older adult (16). Lei et al. (17) synthesized 64 studies to explore the impact of social media use on the psychosocial outcomes of the older adult. While cross-sectional studies generally reported associations between social media use and positive psychosocial outcomes (such as reduced loneliness, depression, anxiety, increased social connections, well-being, life satisfaction, and quality of life), longitudinal studies presented mixed and inconclusive results. This indicated that although social media use was associated with positive psychosocial outcomes, the current lack of consistent conclusions due to methodological and measurement differences called for more standardized and rigorous research to address these inconsistencies (17). Pei et al. (18) compared older adult Chinese individuals in mainland China (Wuhan), Taiwan (Taichung), and the United States (Honolulu) to investigate the relationship among education, social media use, and advance care planning (ACP) discussions. Social media use was positively correlated with ACP discussions in Wuhan and Honolulu and moderated the relationship between education and ACP discussions in Honolulu. This suggested that social media could promote the prevalence of ACP discussions across different cultural contexts, offering new perspectives for policy and practice (18).

The role of social media self-efficacy in the information usage and mental health of the older adult, the barriers and motivators in the use of social technologies, and the relationship between social media use and psychosocial outcomes all provide rich perspectives for research on social media use among the older adult.

## 3 Digital new media art expression and social adaptability

### 3.1 Digital new media art expression

#### 3.1.1 Short videos

**Figure 1:**
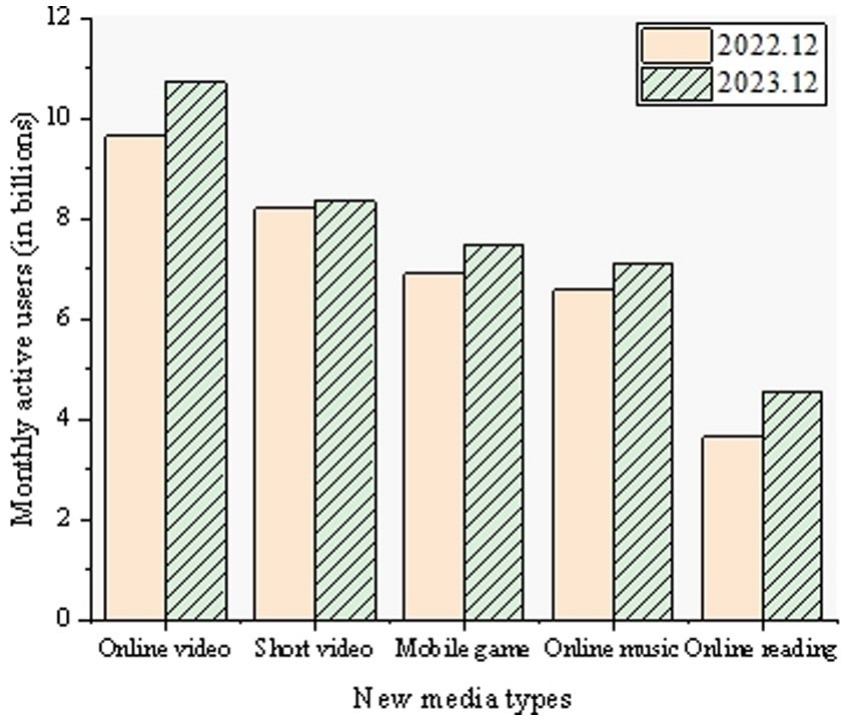
Monthly active users statistics in the short video industry.

Short videos, as a form of brief video content played on digital media platforms, suitable for fragmented time viewing, have amassed a vast user base in China. The monthly active user statistics for the short video industry are depicted in Figure 1.

As of December 2023, the number of short video users in China has reached 1.072 billion, with over half of the population engaging in daily short video consumption. The duration of short videos is relatively brief compared to long-form videos, typically ranging from a few seconds to several minutes. Additionally, the audience spans various age groups, from the older adult to teenagers (19–21). Short videos, owing to their emotional resonance, have captivated widespread attention, leading people to become engrossed and giving rise to a phenomenon known as short video addiction (22). This addictive behavior transcends specific age groups, affecting individuals across all age ranges. Both teenagers and the older adult may find themselves drawn and immersed in short video content. In the context of digital new media art expression driven by AI, short videos possess unique artistic allure. Through short videos, artists can convey rich emotions and thoughts within an extremely short timeframe, touching the deepest resonances within the audience (23). Simultaneously, short videos offer a novel means of artistic expression, enabling artists to flexibly showcase creativity and ideas. However, acceptance levels of short videos may vary among the older adult, a specific demographic. Due to differences in age and cultural background, some older adult individuals may find short video content unfamiliar or challenging to adapt to. Therefore, in digital new media art expression, it is essential to consider the specific needs and acceptance levels of the older adult, providing them with artworks that resonate closely with their lives and emotions (24).

#### 3.1.2 Online financial management

In the realm of digital new media art expression, online financial management, as an application of financial technology, has become a significant form of representation. Through the application of AI technology, online financial management platforms can better provide users with personalized financial plans to meet diverse needs. For the older adult, the emergence of online financial management platforms poses both challenges and opportunities. On the one hand, older adult individuals may experience unfamiliarity and discomfort with digital technology, leading to resistance to using online financial management platforms. On the other hand, with appropriate training and guidance, the older adult can benefit from online financial management platforms, enhancing the efficiency and proficiency of financial management (25). In digital new media art, various methods can be employed to present the theme of online financial management. For example, virtual reality technology can simulate scenes of online financial management, or data visualization can showcase the performance and returns of financial products. These forms of representation not only enhance the artistic quality of the works but also increase the awareness and understanding of online financial management among the older adult (26).

#### 3.1.3 Public account push

In addition to online financial management, public account push is also a significant form of expression in digital new media art. Through public account push, artists can disseminate their works to a broader audience, fostering more direct and intimate interactions with viewers. For older adult individuals, public account push serves as a convenient channel for information retrieval. By following public accounts aligned with their interests, older adult individuals can access the latest art pieces, cultural activities, and other information, enriching their spiritual lives (27). Simultaneously, public account push can act as a bridge for communication between older adult individuals and the younger generation, promoting cultural exchange and understanding across different age groups. In digital new media art, public account push can take various forms, such as text, audio, video, etc. Artists can utilize these formats to showcase their creations, engage in interaction and communication with the audience, thereby enhancing the dissemination and impact of their works.

In summary, online financial management and public account push, as crucial forms of digital new media art expression, are playing an increasingly vital role under the influence of AI. Through these forms, artists can engage in more direct and intimate interactions with the audience, while also fostering social adaptability and cultural exchange among the older adult (28).

### 3.2 Social adaptability

The social adaptability of older adult individuals refers to their ability to adapt to changes in modern society, effectively interact with the environment and others. This concept originates from Darwin’s theory of “survival of the fittest” and has been further defined by scholars such as Heckhausen as the adaptive capacity in human-social interactions. The social adaptability of older adult individuals encompasses aspects such as environmental adaptation, family relationship adaptation, interpersonal communication adaptation, and self-care adaptation (10). With the advent of the information age, the social adaptability of older adult individuals is no longer merely a process of adapting to modern society but rather a functional state in the era of informationization. According to the Law on the Protection of the Rights and Interests of the older adult, older adult individuals have the right to participate in social development, providing legal support for their related rights (14).

**Figure 2:**
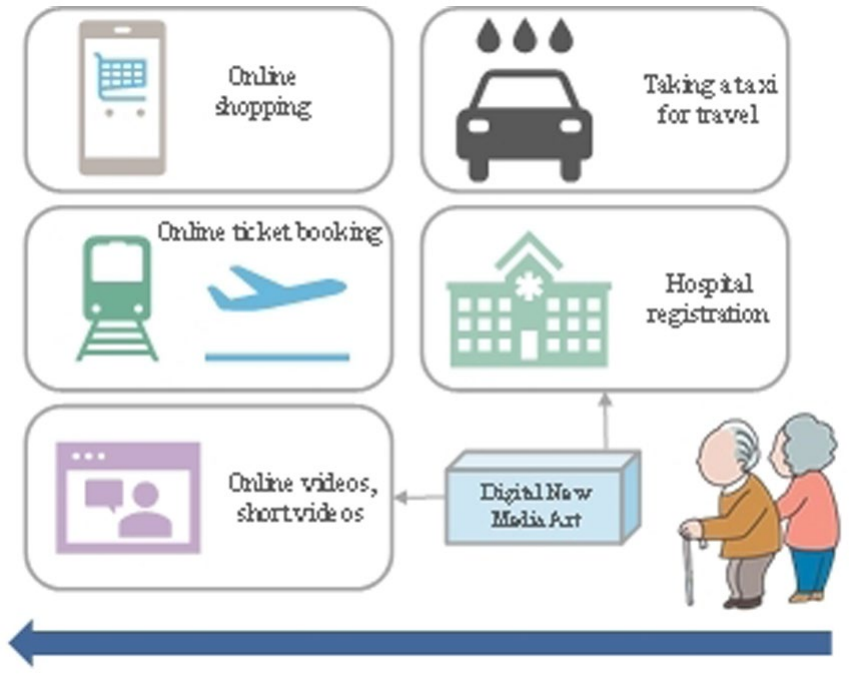
Older adult participation in digital new media.

This study categorizes various material and spiritual activities in which older adult individuals participate as community engagement activities, including older adult education, online entertainment, interpersonal communication, and community information governance. Applying social participation theory, the study advocates for the active involvement of older adult individuals in community activities, facing the challenges of the information age with a positive attitude. The application of social participation theory helps older adult individuals better understand society, unleash the potential in their lives, enhance self-identity and happiness, and also contributes to increasing the cohesion and centripetal force of the community (29). In conclusion, the social adaptability of older adult individuals is not only a process of adapting to modern society but also a functional state in the era of informationization. Through active participation in community activities and the application of social participation theory, older adult individuals can better adapt to modern society, unleash their own value, enhance happiness, and contribute to community cohesion (30). In the context of digital new media art expression driven by AI, the typical participation modes of older adult individuals in digital new media are illustrated in Figure 2.

AI is primarily connected to digital new media art and the social adaptability of the older adult through the following means: (a) AI in the Creation and Recommendation of Digital New Media Content: AI algorithms can generate short videos, art, music, and other multimedia content, enriching the cultural and spiritual lives of the older adult. Recommendation systems use AI technology to suggest personalized content, such as news, entertainment videos, and health information, based on the interests and behavioral data of the older adult. This facilitates easier access to information, enhancing their quality of life. (b) AI-Driven Voice Assistants (e.g., Alexa, Siri) and Smart Home Devices: These technologies provide convenient services for the older adult. Through voice commands, seniors can easily access information, set reminders, play music, or control smart devices at home. These advancements help the older adult better adapt to modern life, boosting their independence and confidence. (c) Social Media and Virtual Communities: AI applications on social media platforms, such as intelligent matching and friend recommendations, as well as the analysis of user emotions and behaviors, can enhance the interactivity and sense of participation of the older adult in virtual communities. These platforms enable seniors to stay connected with family and friends and engage in various online activities, thereby reducing loneliness and strengthening social connections and adaptability. (d) Online Education and Health Management: AI applications in online education and health management can help the older adult acquire new knowledge and skills, improving their digital literacy. AI can offer personalized online courses and learning plans, assisting the older adult in learning to use smart devices and digital tools. Additionally, health management applications utilizing AI technology can monitor seniors’ health data, providing health advice and reminders to promote their physical health and psychological well-being. e.g. Digital Art Creation Tools: AI-driven digital art creation tools, such as image generation and music composition software, enable the older adult to engage in artistic activities more easily. This not only enriches their leisure time but also stimulates their creativity and sense of accomplishment, enhancing their self-identity and social adaptability.

Through these various methods, AI advances the development of digital new media art and plays a crucial role in enhancing the social adaptability of the older adult. The application of AI technology in digital new media art not only enriches the spiritual and cultural lives of seniors but also provides more opportunities for social interaction and learning, facilitating better adaptation to the changes in modern society.

### 3.3 Semi-structured interviews

In this study, a total of 10 older adult individuals were interviewed, with 5 from Community *A* and 5 from Community *B*. The selection aimed to encompass a broad age range, varied educational backgrounds, and diverse occupational experiences during their youth. Basic personal information about the older adult participants included their community of residence, age, education level, former workplace, position, and a brief overview of their family situation. Specific details are presented in Table 1.

**Table 1:** Basic personal information of older adult participants.

Number	Name	Age	Education	Former workplace	Position	Family
Community *A*						
1	Respondent A	70	Junior high school	Cultural center	Artist	Living with son
2	Respondent B	65	Junior High school	Library	Librarian	Living alone
3	Respondent C	72	Elementary school	Hospital	Nurse	Living with husband
4	Respondent D	68	Junior high school	Post office	Mailman	Living with wife
5	Respondent E	73	College degree	Cultural bureau	Cultural Heritage Inheritor	Living alone
Community *B*						
6	Respondent F	71	Elementary school	A high school	Math teacher	Living with son
7	Respondent G	70	University degree	Beijing Jiaotong University	Professor	Living with wife
8	Respondent H	66	Technical secondary school	Dongsheng township government	Finance	Living alone
9	Respondent I	69	Technical secondary school	Industrial bureau, former wire rope factory, former shoe factory	Secretary	Living with wife
10	Respondent J	64	University degree	Team accountant, former shoe factory	Accountant, Worker	Living alone

Interviews are conducted with four staff members from the two communities, aiming to understand the basic situation of the communities. This is done to ensure that the staff members have sufficient knowledge and work experience related to the community, thereby ensuring the basic validity and reliability of the study. After obtaining a preliminary understanding of the basic background of the community staff, interviews are conducted to investigate the relevant path choices in the process of community informatization governance construction. Basic information covers educational background, position, working hours, and responsibilities. An overview of the basic information of staff members from Communities *A* and *B* is presented in Table 2.

**Table 2:** Basic information of staff members from communities *A* and *B*.

Number		Name	Age	Education	Position	Work experience	Work responsibilities
1	Community *A*	Staff K	52	College degree	Director of *A* Community Residents’ Committee	16 years	Liaison with the sub-district office, issuance of various specific tasks within the community.
2		Staff L	30	Bachelor’s degree	Deputy Director of *A* Community Residents’ Committee	12 years	Coordination and supervision of daily community work, online dissemination, organizing older adult activities.
3	Community *B*	Staff M	46	College degree	Director of *B* Community Residents’ Committee	17 years	Liaison with the sub-district office, issuance of various specific tasks within the community.
4		Staff N	33	Bachelor’s degree	Deputy Director of *B* Community Residents’ Committee	12 years	Coordination and supervision of daily community work, organizing older adult activities, managing community public account promotion, etc.

Both Community *A* and Community *B* are located in urban areas of China, representing different types of urban communities. Community *A* is a more traditional community, primarily inhabited by local older adult residents. It boasts well-developed community service facilities and frequent interactions among residents. In contrast, Community *B* is an emerging community, with residents comprising both locals and migrant workers. Although Community *B* is developing rapidly, its older adult services are still being improved. These two communities are typical and representative in terms of older adult social adaptability and the application of digital new media art. Participants are invited with the assistance of community committees, ensuring the diversity and representativeness of the sample.

The study selects older adult participants from various age groups, educational backgrounds, occupational histories, and family structures to comprehensively understand the acceptance of digital new media art and social adaptability among seniors from different backgrounds. The selection process adheres strictly to the research design, ensuring that the sample accurately represents the diversity of older adult residents in the community. Semi-structured interviews with open-ended questions are conducted to encourage respondents to freely express their views on digital new media art and social adaptability. The interview questions cover topics such as the use of smartphones and digital new media art, the frequency and experiences of participating in community activities, the main channels of information acquisition, the choice of entertainment activities, and the use of mobile payment functions. This approach provides in-depth insights into the actual needs and challenges faced by the older adult, thereby offering a scientific basis for enhancing their social adaptability.

## 4 Older adult individuals’ use of smart facilities

### 4.1 Acceptance of new media art among older adult community

In this study, semi-structured in-depth interviews were conducted with five older adult individuals and two community workers from Community *A*, as well as five older adult individuals and two community workers from Community *B*. By understanding the basic information of older adult individuals and community workers, along with the approach to community informatization governance, the study summarizes the adaptability of older adult residents in dealing with new media art products, community smart facilities, and information management. The selection paths for community informatization are explored, and a comparative analysis is made between the two communities. The acceptance of new media art among older adult community residents is outlined in Table 3.

**Table 3:** Acceptance of new media art among older adult community residents.

Number	Community	Name	Computer exposure in previous job	Smartphone usage	Participation in community activities	Exposure to digital new media art
1	Community *A*	Respondent A	Yes	Proficient in most functions	Yes	Yes
2		Respondent B	No	Basic usage of some functions	No	Yes
3		Respondent C	Yes	Proficient in most functions	Yes	Yes
4		Respondent D	No	Doesn’t use	No	No
5		Respondent E	Yes	Proficient in most functions	Yes	Yes
6	Community *B*	Respondent F	Yes	Doesn’t use	No	No
7		Respondent G	No	Basic usage of some functions	Yes	Yes
8		Respondent H	No	Doesn’t use	Yes	No
9		Respondent I	No	Doesn’t use	Yes	Yes
10		Respondent J	No	Doesn’t use	Yes	No

Through interviews, there are notable differences between the older adult residents of Community *A* and Community *B* in terms of the use of smartphones, participation in community activities, and the utilization of community basic information devices. As indicated by the data in Table 3, the usage of smartphones by older adult residents is closely related to their prior exposure to computers during their working years. With increasing age, the older adult exhibit a gradual decrease in their acceptance of new technologies, resulting in a corresponding decline in their social adaptability. However, it was also observed that if older adult individuals had previous exposure to computers in their younger years, their subsequent use of smartphones might be more seamless. Notably, in Community *A*, the involvement of older adult residents in the community volunteer WeChat team, facilitated through the use of smartphones, significantly enhances their level of community engagement. This mode of participation in digital new media art holds significant meaning for the older adult, not only integrating them more deeply into the community but also facilitating better communication and interaction with the younger generation.

To substantiate the findings that older adult residents in Community *B* have a lower acceptance of digital new media art and a less significant correlation with community activity participation, this study conducts statistical analyses comparing various dimensions between Community *A* and Community *B*. These dimensions include the frequency of community activity participation, the acceptance of digital new media art, and the correlation between these factors. The statistical analysis of older adult residents’ acceptance of digital new media art and participation in community activities in Communities *A* and *B* is presented in Table 4.

**Table 4:** Statistical analysis of older adult residents’ acceptance of digital new media art and participation in community activities in communities *A* and *B.*

Project/community	A community (*N* = 5)	B community (*N* = 5)	Statistical significance (*p*-value)
Frequency of participating in community activities (times/month)	4.2 ± 1.3	2.6 ± 1.1	0.03
Digital new media art acceptance score (1–5)	4.0 ± 0.8	2.5 ± 0.7	0.01
Offline community activity participation rate (%)	60%	80%	0.15
Online community activity participation rate (%)	80%	40%	0.04
Correlation between digital new media art and community activities	Strong correlation (*r* = 0.75)	Weak correlation (*r* = 0.30)	0.02

In contrast, community activities in Community *B* predominantly take place offline, and the older adult residents demonstrate a relatively lower acceptance of digital new media art. Consequently, their correlation with participation in community activities is not as pronounced. This suggests that when promoting the application of digital new media art among the older adult, targeted planning and implementation should be carried out based on the characteristics of different communities and the actual circumstances of the older adult residents.

### 4.2 Smartphone usage among older adult community residents

The social adaptability of older adult individuals encompasses their ability to adapt to fresh changes, adjust to shifts in social roles, and cope with psychological and physiological changes resulting from their own physical conditions. Given the varying degrees of social adaptability among the older adult, community governance should make diverse choices in response. To better summarize the extent of social adaptability among the older adult, this study takes the example of smart device usage, exploring the relationship between digital new media art expression and the social adaptability of older adult individuals. The study surveyed 10 older adult individuals, categorizing the investigation into five major aspects: interpersonal communication, information acquisition, entertainment, practical applications, and mobile payment functions. Through interviews and observations, the study explored whether older adult individuals meet these needs in their daily lives through smartphone usage and the difficulties and challenges they encounter when facing new technology. The aim is to understand the usage patterns of smartphones among older adult individuals, their performance in social interactions, and further evaluate their social adaptability. Detailed survey results for Community *A* are provided in Table 5.

**Table 5:** Detailed survey results for community *A*.

Interviewee	Social interaction function	Information retrieval function	Entertainment function	Practical application function	Mobile payment function
Respondent A	WeChat messaging, WeChat video, news viewing	Browsing websites (e.g., Baidu), Watching videos	Gaming	Beijing Health Treasure (health management), appointment scheduling	Online financial management, QR code payment, online shopping
Respondent B	WeChat messaging, WeChat video, news viewing	Browsing websites (e.g., Baidu)	–	–	–
Respondent C	WeChat messaging, WeChat video, news viewing	Browsing websites (e.g., Baidu), watching videos, gaming	Beijing Health Treasure (health management), appointment scheduling	Online financial management, QR code payment, online shopping	–
Respondent D	WeChat messaging, WeChat video, news viewing	Browsing websites (e.g., Baidu), watching videos	Gaming	Beijing Health Treasure (health management), appointment scheduling	Online financial management, QR code payment, online shopping
Respondent E	WeChat messaging, WeChat video, news viewing	Browsing websites (e.g., Baidu), watching videos	Gaming	–	–

Through a survey of 10 older adult individuals in Community *A*, it was observed that their performance in smart device usage varied. Among them, Mrs. Wang, Mrs. Yang, Mrs. Zhang, Mr. Chen, and Mr. Zhou were all proficient in using WeChat for message exchange, video calls, and news browsing. They could also utilize search engines like Baidu to look up information on websites. Some of these older adult individuals also enjoyed entertainment activities on their phones, such as playing games or watching videos. In practical applications, they were accustomed to using Beijing Health Treasure for health management and appointment scheduling. Additionally, they engaged in online financial management, QR code payments, and online shopping operations. However, there were also some older adult individuals lacking in certain functions. For instance, Mrs. Yang and Mr. Zhou were more conservative in their approach to entertainment functions, not exploring games or other recreational activities. Mrs. Zhang, on the other hand, had certain gaps in practical applications. These differences might be influenced by individual interests, health conditions, and the level of acceptance of new technologies. In summary, the social adaptability of the older adult is reflected to a certain extent in their usage of smart devices. Through exploration and application of different functions, older adult individuals demonstrate a positive learning attitude and adaptability, while also highlighting individual variations.

Detailed survey results for Community *B* can be found in Table 6.

**Table 6:** Detailed survey results for community *B*.

Interviewee	Social interaction function	Information retrieval function	Entertainment function	Practical application function	Mobile payment function
Respondent F	WeChat messaging, WeChat video, news viewing	Browsing websites (e.g., Baidu), watching videos	Watching videos	Beijing Health Treasure (health management)	–
Respondent G	–	–	–	–	–
Respondent H	–	–	–	–	–
Respondent I	–	–	–	–	–
Respondent J	WeChat video	Reading news	Watching videos	Beijing Health Treasure (health management)	–

The survey on the use of smartphone functions by older adult individuals in Community *B* clearly indicates that they use these functions less compared to the older adult in Community *A*. This difference may be related to community characteristics, such as community type, regional economic foundation, familiarity among residents, and the services provided by the community. The dependence on people around them is an important factor influencing the use of smartphones by the older adult. In addition, the level of familiarity among community residents and social needs affect the older adult’s use of smartphones. Compared to Community *A*, residents in Community *B* are more familiar with each other, engage in more frequent social activities, and Community *B* also offers more offline activities and services tailored for the older adult, enriching the daily lives of the older adult and meeting their social interaction needs. Therefore, the social adaptability of the older adult in Community *B* shows some differences.

Mrs. Wu is the only respondent in Community *B* who applies smartphone functions. She can proficiently use WeChat for messaging and video calls, search engines like Baidu for website information, and also enjoys watching videos. In terms of practical applications, she is accustomed to using Beijing Health Treasure for health management. On the contrary, other respondents such as Mr. Zheng, Mrs. Tang, and Mrs. Liu did not show obvious signs of use in various functions. Mr. Han has some application in certain functions, but overall, he did not demonstrate a high level of usage. This difference may be influenced by individual interests, the level of acceptance of new technologies, and health conditions.

The significance of this study lies in its exploration of the impact of AI-driven digital new media art on the social adaptation of the older adult. The importance of this research is highlighted in the following aspects, underscoring its practical implications for policymakers, healthcare professionals, and other stakeholders involved with the older adult: (a) Enhancement of older adult Social Adaptation: By investigating the effects of AI-driven digital new media art on the older adult, this study aims to understand the role of these emerging technologies in enhancing social adaptation among seniors. The findings may reveal positive impacts of digital new media art on aspects such as community participation, information acquisition, entertainment, and practical applications, thus providing a robust empirical basis for improving older adult social adaptation. (b) Basis for older adult Health Policy Development: With the intensifying trend of population aging, older adult health has become a key societal focus. This study can inform the formulation of relevant policies by demonstrating the impact of AI-driven digital new media art on older adult social adaptation. Policymakers can leverage these findings to adjust policies, promoting the integration of digital new media art within the older adult demographic, thereby facilitating social inclusion and healthy aging. (c) Guidance for Healthcare Professionals: Studying the application of digital new media art among the older adult offers valuable insights for healthcare professionals. It guides them in clinical practice to better utilize these technologies and resources, enhancing both the physical and mental well-being of the older adult. Additionally, healthcare institutions can reference these findings to innovate and optimize older adult health services. (d) Reference for Other Stakeholders: Beyond policymakers and healthcare professionals, other stakeholders interacting with the older adult—such as community organizations, social workers, and family members—can also gain insights and guidance from this research. They can develop relevant social service projects or provide support tailored to the needs of the older adult, promoting their comprehensive development and well-being.

Therefore, the results of this study hold significant practical implications for policymakers, healthcare professionals, and other stakeholders involved with the older adult. These findings can guide better attention and support for the health and happiness of the older adult.

## 5 Conclusion

This study aims to explore the impact of AI-driven digital new media art on the social adaptability of older adult individuals. Through interviews and observations of older adult individuals in Communities A and B, the study discusses the application of digital new media art in the older adult population. Based on the survey results, the following conclusions can be drawn. Firstly, there are differences in the application of digital new media art in the older adult population. Older adult individuals who have previous computer experience in their work are more likely to accept smartphones and actively participate in community activities. Digital new media art significantly enhances the level of community involvement among the older adult, promoting community vitality and cohesion. However, the acceptance of digital new media art varies among older adult individuals in different communities, requiring personalized promotion strategies based on specific circumstances. Secondly, there are notable differences in the usage levels of digital new media among the older adult. Some older adult individuals can proficiently use various functions, while others are relatively conservative. Digital new media art enriches the cultural and spiritual lives of the older adult, enhancing their social adaptability. However, there is a need to further improve the acceptance of digital technology among the older adult to better adapt to the development of a digitized society.

Although this study provides an in-depth exploration of the social adaptability of older adult individuals in the era of digital new media, it has certain limitations. The study mainly focuses on the impact of digital new media art expression on the social adaptability of the older adult, and there is insufficient exploration of the specific influencing mechanisms. Future research could employ experimental designs or longitudinal methods to further investigate the specific mechanisms through which digital new media art expression affects the social adaptability of older adult individuals.

## Data Availability

The original contributions presented in the study are included in the article/supplementary material, further inquiries can be directed to the corresponding author.
